# KMWin – A Convenient Tool for Graphical Presentation of Results from Kaplan-Meier Survival Time Analysis

**DOI:** 10.1371/journal.pone.0038960

**Published:** 2012-06-15

**Authors:** Arnd Gross, Marita Ziepert, Markus Scholz

**Affiliations:** 1 Institute for Medical Informatics, Statistics and Epidemiology, University of Leipzig, Leipzig, Germany; 2 LIFE – Leipzig Research Center for Civilization Diseases, University of Leipzig, Leipzig, Germany; Istituto Superiore di Sanità, Italy

## Abstract

**Background:**

Analysis of clinical studies often necessitates multiple graphical representations of the results. Many professional software packages are available for this purpose. Most packages are either only commercially available or hard to use especially if one aims to generate or customize a huge number of similar graphical outputs. We developed a new, freely available software tool called KMWin (Kaplan-Meier for Windows) facilitating Kaplan-Meier survival time analysis. KMWin is based on the statistical software environment R and provides an easy to use graphical interface. Survival time data can be supplied as SPSS (sav), SAS export (xpt) or text file (dat), which is also a common export format of other applications such as Excel. Figures can directly be exported in any graphical file format supported by R.

**Results:**

On the basis of a working example, we demonstrate how to use KMWin and present its main functions. We show how to control the interface, customize the graphical output, and analyse survival time data. A number of comparisons are performed between KMWin and SPSS regarding graphical output, statistical output, data management and development. Although the general functionality of SPSS is larger, KMWin comprises a number of features useful for survival time analysis in clinical trials and other applications. These are for example number of cases and number of cases under risk within the figure or provision of a queue system for repetitive analyses of updated data sets. Moreover, major adjustments of graphical settings can be performed easily on a single window.

**Conclusions:**

We conclude that our tool is well suited and convenient for repetitive analyses of survival time data. It can be used by non-statisticians and provides often used functions as well as functions which are not supplied by standard software packages. The software is routinely applied in several clinical study groups.

## Introduction

Clinical studies often necessitate repetitive data analyses and the need of multiple graphical representations of the results. Presentations and publications often have different requirements regarding graphical output styles. Hence, series of analyses and corresponding graphical outputs need to be adapted frequently. Many professional software packages for data analysis and graphical representation are available such as SPSS [1], SAS [2] or R [3], [4]. These packages are either only commercially available or do not satisfy certain needs, such as displaying number of cases under risk and number of cases within the graphical output, inversion of survival time curves or adjustment of p-value accuracy. Additionally, these packages are complicated to use for non-statisticians, especially if one aims to construct series of high-quality figures. In this context “complicated” means either providing a huge functionality attained by a confusing number of menus (e.g. SPSS) or offering hardly more than a console which requires knowledge of suitable routines and syntax to manipulate graphical settings (e.g. R, SAS).

We developed the new software tool KMWin (Kaplan-Meier for Windows) for graphical presentation of results from Kaplan-Meier survival time analysis. It combines both, free availability and provision of an easy to use interface. The interface comprises often used functions and features, which are not supplied by standard software packages. The functionality of the programme was developed in close cooperation with biometricians and trial assistants who are involved in analysing numerous large multi-centre studies encompassing survival time analysis, e.g. [5]–[7].

In the present paper, we demonstrate how to use KMWin and present its main functions on a working example. We show how to control the interface, customize the graphical output, calculate statistical tests and filter and reanalyse survival time data. Furthermore, we compare KMWin with SPSS with respect to graphical output, statistical output, data management and development of the software.

## Results

### Overview of Functionality

Kaplan-Meier analysis and drawing the corresponding survival curves are achieved by controlling the statistical software environment R [3], [4]. That is, KMWin is an interface and intended to make working with R easier.

Our software provides all functions which are needed to create survival time curves in a simple manner allowing various features. When loading KMWin, it tries to locate R, invokes it and establishes the connection. The status bar of the programme is located at the bottom of the window and assists in creating plots by displaying hints. A comprehensive HTML help document is included and the appropriate section of the document is displayed when it is called within the programme. Hotkeys are provided to speed up input.

KMWin consists of different windows to manipulate the settings. Five windows are available and described below. The items on each window of KMWin are explained in greater detail in the HTML Help (included in Package S1).

#### Main window

**Figure 1:**
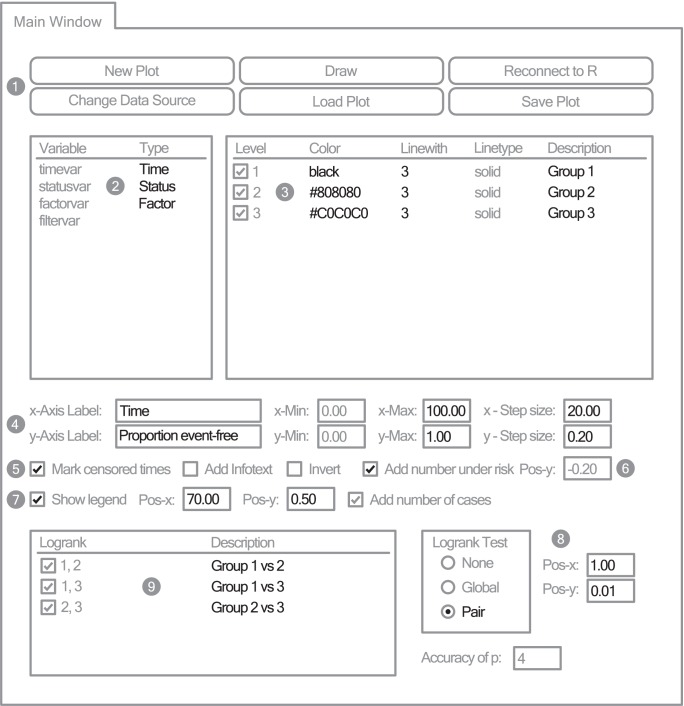
KMWin Main Window. Simplified representation of KMWin Main Window. Settings apart from default are black. Filled grey circles are labels for: 1-Button area, 2-Variables table, 3-Factor levels table, 4-Axes settings, 5-Censored times setting, 6-Number under risk settings, 7-Legend settings, 8-Logrank test settings and 9-Logrank table.

Plots are managed on the Main Window (Figure 1). Particularly, plots can be created, stored into files and loaded (Label 1 in Figure 1). Variables of the data source are listed in a table. A time, status and optionally a factor attribute can be assigned to these variables (Label 2 in Figure 1). Plot properties such as colour, line style and legend text can be modified here. If a factor variable is chosen, then the plot properties of each factor level could be changed (Label 3 in Figure 1). Further adjustments are possible such as adaption of axes limits and labels (Label 4 in Figure 1), marking censored times (Label 5 in Figure 1), adding an information text or a legend to the plot (Label 7 in Figure 1), inverting the survival time curve, displaying a table with cases under risk at the respective time steps (Label 6 in Figure 1) or performing Logrank Tests across strata with desired p-value accuracy (Label 8 in Figure 1).

#### Filter window

**Figure 2:**
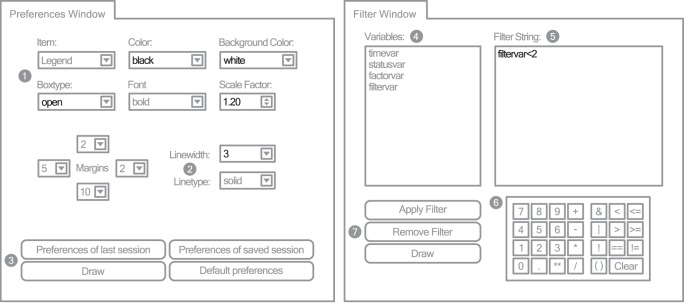
KMWin Preferences Window (left) and Filter Window (right). Simplified representation of KMWin Preferences Window and Filter Window. Settings apart from default are black. Filled grey circles are labels for: 1-Item properties, 2-General line properties, 3-Button area, 4-Variables table, 5-Filter string edit box, 6-Input panel, 7-Button area.

Filtering survival time data prior to analysis can be achieved by applying a filter string (Figure 2). Filter strings are made of appropriate concatenation of variables, logical operators, mathematical operators and bracketing. Strings which are not syntactically correct or which filter out all data were caught by the software.

#### Preferences window

All general settings apart from curve selection and other curve properties may be altered on the Preferences Window (Figure 2). These preferences comprise adapting colours, frames, fonts, and scaling of different components like the plain, axes, labels, text boxes or tables. The size of margins of the plot area and the general width and line type of all objects can be adjusted here. The most recent preferences are automatically saved between sessions.

#### File queue window

**Figure 3:**
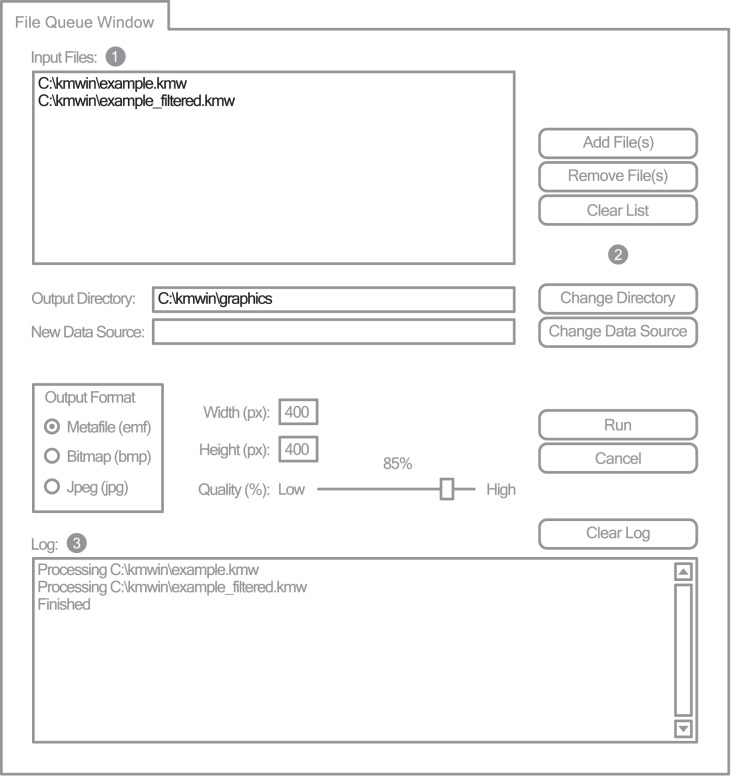
KMWin File Queue Window. Simplified representation of KMWin File Queue Window. Settings apart from default are black. Filled grey circles are labels for: 1-Input files table, 2-Button area, 3-Log text box.

If one likes to re-evaluate a number of previously created plots, they could be added to the file queue (Figure 3). The queue is a list of plots which are sequentially processed and saved in different output formats. KMWin currently supports generating emf, bmp and jpg files.

#### Log window

**Figure 4:**
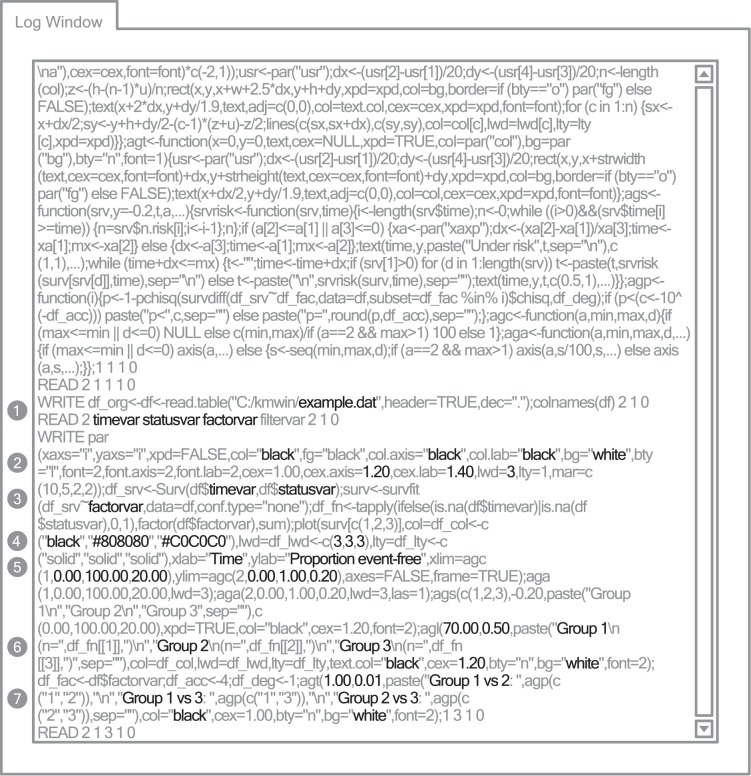
KMWin Log Window. Simplified representation of KMWin Log Window. Black lettering emphasizes user settings embedded in R commands. Filled grey circles are labels for: 1-Sample write and read command (load survival data and request variable names), 2-Settings for colours of plain, axes and labels; scaling of axes and labels; general line width, 3-Survival time analysis with user definied variables timevar, statusvar and factorvar, 4-Colours and line widths of survival curves, 5-Axes settings, 6-Legend settings, 7-Logrank table settings.

Each step of Kaplan-Meier survival time analysis managed by KMWin can be tracked as a sequence of R commands, beginning with data import, selection of variables, changing curve properties and so on until finally plotting the results. Every command which was sent to R can be viewed on the Log Window (Figure 4).

### Working Example

**Figure 5:**
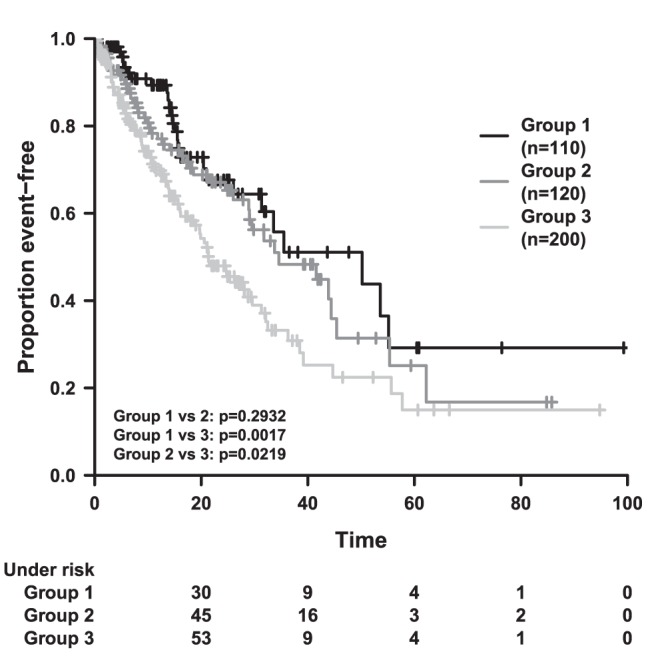
Survival Time plot corresponding to unfiltered sample data.

In the following, we analyse the example data included in Package S1, demonstrating the functionality of the Main Window, Preferences Window, Filter Window, File Queue Window and Log Window. When running KMWin, it tries to locate and run R automatically. If this fails, the connection can be established by first running R and second KMWin. In Figure 1 one can find a simplified representation of the Main Window. A new plot can be created by using the “New Plot” button which is located at the Button area on the Main Window (Label 1 in Figure 1). When the “Data Source” window appears, set the Data type to ASCII (*.dat) and select “example.dat” which is included in our software package. By right clicking within the “Type” column of the Variables table (Label 2 in Figure 1) one can select the time, status and (optional) factor variable for survival time analysis. A meaningful selection is “timevar” as time variable, “statusvar” as status variable and “factorvar” as factor variable. In general, properties may be modified by right clicking within the right column of the corresponding table. The “Draw” Button (Label 1 in Figure 1) may be used to create or update the graphical output of the analysis. At this stage, the output differs from Figure 5 which we will try to construct. For example, the default colour scheme was made for coloured slides, but for our analysis we would prefer results in greyscale. The colour of the survival curve corresponding to each factor level can be changed in the Factor levels table (Label 3 in Figure 1). For level 1 we choose “black” which is one of the default colours. For levels 2 and 3 we choose “User defined” colours with RGB values (Red: 128, Green: 128, Blue: 128) for “dark grey” and (Red: 192, Green: 192, Blue: 192) for “light grey”, respectively. KMWin displays the hexadecimal notation #808080 and #C0C0C0 for level 2 and 3. This is common for user defined colours (e.g. in HTML). We prefer greater line width, so change “Linewidth” to 3 for each of the three levels. Furthermore, we change the curve description which defaults to “factorvar = i” for each level i of the factor “factorvar”. By double left or right clicking, we update the descriptions of level i = 1,2,3 to “Group 1″, “Group 2″ and “Group 3″, respectively and press Enter key. These curve descriptions belong to the legend of the figure. Furthermore, we want to replace the default axes labels of the x-Axis and y-Axis by “Time” and by “Proportion event-free”, respectively (Label 4 in Figure 1). If the maximum of an axis is defined equal (default) or less than the minimum or the step size is equal or less than zero, the scaling is performed by internal rules of the plot function of R. We prefer stringent limits and set “x-Min”, “x-Max”, “x-Step size” to 0, 100, 20 and “y-Min”, “y-Max”, “y-Step size” to 0, 1 and 0.2, respectively. We also wish to mark censored times (Label 5 in Figure 1) and want to add numbers under risk (Label 6 in Figure 1). Moreover, we tick “Show legend” and set the lower left coordinate to (Pos-x: 70, Pos-y: 0.5) (Label 7 in Figure 1). Finally, we enable the output of p-values corresponding to all pair-wise tests of survival time curves against the null hypothesis of equality by choosing the pair-wise Logrank test and set the lower left coordinate of the corresponding text box to (Pos-x: 1,Pos-y: 0.01) (Label 8 in Figure 1).

The default description of pair-wise tests is “factorvar = i, j” for comparison of factor level i = 1,2,3 with level j = i+1,…,3. By editing the description field of the table of tests (Label 9 in Figure 1), we change the descriptions to “Group 1 vs 2″, “Group 1 vs 3″, “Group 2 vs 3″ for comparison of level 1 with level 2, level 1 with level 3 and level 2 with level 3, respectively by double left or right clicking and finishing with Enter key. The accuracy of p-values is set on four and can be changed if desired.

Only a few more colour and style changes have to be made to achieve a graphical output identical to Figure 5. General colour and style settings apart from curve properties can be modified on the Preferences Window as depicted in Figure 2. We will adapt the properties of different items such as the Plain (refers to the coordinate system), Axes (numbers at the axes), Label (axes label), Legend, Logrank table and the table with cases under risk (see Label 1 in Figure 2). Properties which are not available for the corresponding item are greyed out. For each of the seven items, we change “Color” to “black” and “Background color” to “white”. Additionally, we remove the surrounding boxes for the items “Plain”, “Legend” and “Logrank” by setting the “Boxtype” to “open”. Next, we adjust the font scaling, and set the “Scale factor” for the item “Axes” to 1.2, for the item “Label” to 1.4 and for the items “Legend” and “Under risk” to 1.2. Finally, we increase the general line width to 3 for all lines apart from survival curves (Label 2 in Figure 2). The effect of each modification can be checked by using “Draw” which is located in the Button area (Label 3 in Figure 2). Now, the graphical output is supposed to be identical to Figure 5. The graphical output can be saved to a file by right clicking within the R window or by utilizing R’s File menu. Changes made to KMWin should now be saved to a file (e.g. “example.kmw”) by clicking the “Save plot” button on the Main Window (Label 1 in Figure 1).

Next, we demonstrate the use of filters aiming to remove the set of N = 70 individuals with higher risk from our sample. These individuals are indicated by “filtervar = 2″ in contrast to “filtervar = 1″ which indicates the other individuals. Filters can be set on the Filter Window (Figure 2). By double clicking the left mouse key, we select “filtervar” from the Variables table (Label 4 in Figure 2) and enter “filtervar<2″ to the Filter string edit box (Label 5 in Figure 2). The filter string can be entered either by typing it in or by utilizing the Variables table and the Input panel (Label 6 in Figure 2). The input panel of this window comprises numbers and logical symbols similar to SPSS. For details see the HTML help. The filter is applied by using “Apply Filter” in the Button area (Label 7 in Figure 2) and corresponding graphical results can be generated by “Draw” allowing assessment of the differences to the non-filtered analysis immediately.

**Figure 6:**
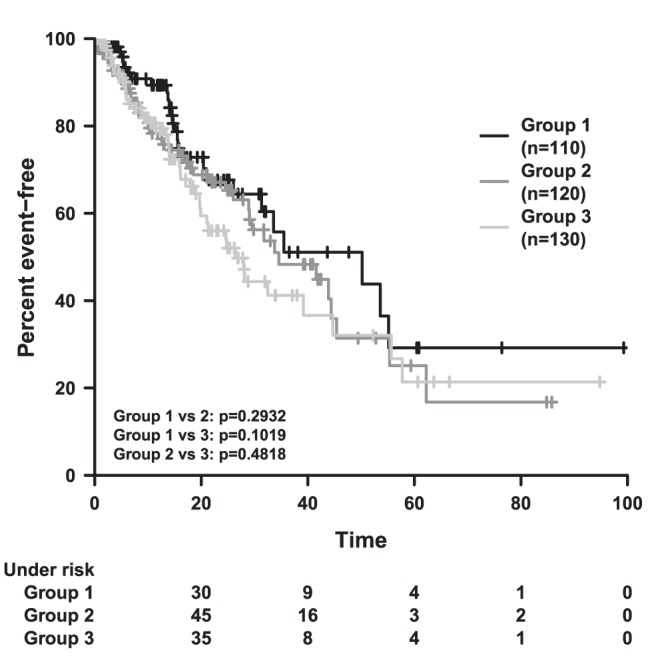
Survival Time plot corresponding to filtered sample data.

The y-axis should be in units of percentage. Hence, the “y-Max” value is changed to 100 and the “y-Step size” to 20 in the Axes settings on the Main Window (see Label 4 in Figure 1). If a “y-Max” value greater than 1 is specified, the unit of y-Axis is automatically interpreted as percentage. For consistency, the y-Axis label should be updated to “Percent event-free”. The graphical result is shown in Figure 6. Now all changes should be saved to “example_filtered.kmw” by using “Save Plot” (Label 1 in Figure 1).

Finally, we demonstrate how analyses can be quickly repeated utilizing the File Queue Window (Figure 3). The Input files table (Label 1 in Figure 3) shows a list of plots which should be processed. Add both previously saved plots (“example.kmw”, “example_filtered.kmw”) to this list by using “Add File(s)” which is located on the Button area (Label 2 in Figure 3). Select an output directory for the graphical results by clicking “Change directory” and initiate process using “Run”. The Log text box (Label 3 in Figure 3) shows all files which are being processed, and if applicable, errors. The key word “Finished” signals the end of processing. Since the default output format “Metafile (emf)” was selected, two files “example.emf” and “example_filtered.emf” are created.

The communication between KMWin and R can be monitored on the Log Window (see Figure 4). Briefly, instructions sent to R are below the keyword “WRITE”, e.g. loading survival time data from file and requesting variable names. Answers from R are below the keyword “READ”, e.g. variable names of that file (Label 1 in Figure 4). Figure 4 presents even more examples how user settings are embedded in R instructions (Label 2–7). More details can be found in the HTML help.

### Comparison with SPSS

SPSS is a commonly used software package in statistics providing different kinds of survival time analysis. Since it is relatively easy to handle, it is suitable for comparison with KMWin. We assess the functions of KMWin in comparison to the most recent available version of SPSS (release 20.0.0). We are interested in similarities and differences regarding the categories “Graphical Output”, “Statistical Output”, “Data Management” and “Development”. “Graphical Output” refers to the modification of colour, style and scaling of different components. “Statistical Output” relates to statistics results from survival time analysis presented at the figure. The category “Data Management” addresses input files, output files and the re-evaluation of analyses. “Development” gives attention to the development of the software and the relation between developer and user.

#### Graphical Output

Almost all objects in survival time plots created with SPSS can be manipulated regarding position, style and colour by cumbersome clicking on objects. In contrast, KMWin provides a straightforward set of options and preferences. Therefore, KMWin is not as versatile as SPSS, but the smaller number of choices speeds up customization of the graphical output. The export of vector graphics is important for refining the results, e.g. for publications. In the early days in 2004, when KMWin was designed, SPSS did not provide satisfying graphical outputs resulting from survival time analysis. Nowadays, SPSS supports the export of vector graphics as KMWin does (via R). However, the SPSS vector graphic export brings inconveniences as text is made of polygons and can therefore hardly be adapted.

#### Statistical Output

As “Graphical Output”, SPSS provides more statistical results than KMWin, especially different statistical tests are available for testing survival times regarding a factor. KMWin only provides the Logrank Test, but can display p-values with adjustable accuracy within the figure. Additionally, it can display number of cases and number of cases under risk at certain time points within the figure. The latter is useful for interpretation of Kaplan-Meier curves but not available in SPSS.

#### Data Management

SPSS allows several input formats while KMWin supports only text files, SPSS files and SAS files for input, which are most often used when we deal with survival time data. The statistical output of SPSS is written to a standardized Output window and from there, the results can be exported to various formats. On the other hand, KMWin plots the graphical result in an R window, where it might be copied to the clipboard or saved as a file in every format supported by R. Using SPSS, repeated interim analyses can be done by running an appropriate script and evaluating the Output window. Knowledge of scripting is not necessary when using KMWin. Saved plots can simply be added to the File queue. Processing the queue generates a graphic file for each queue entry.

### Development

Software is typically not free of problems (software bugs) which are caused by unforeseen states of the software. If someone experiences problems regarding a commercial software package sold to many customers such as SPSS, then the Support is responsible for managing further steps. Sooner or later, the problem will be solved by suggesting a work-around or providing a software patch. In contrast, if the software is less complex like KMWin, problems can be solved easier by communicating the issue directly to the developer. Also improvement of the software is achieved much more straightforward by cooperation of the developer and the user, who experienced a problem or is interested in innovations.

## Discussion

In order to facilitate repetitive analyses of survival data in clinical trials, we constructed the software tool KMWin which is based on R but could be handled easily by clinical trial assistants or data managers without programming skills and knowledge of R syntax. In the present paper, we demonstrated the functionality of KMWin on the basis of simulated survival data. We compared the graphical outputs of KMWin with those of SPSS, which is a popular software for statistical analyses. KMWin does not offer as much options as SPSS to customize the graphical output, but it comprises often used functions in one window which helps to speed up the development process. Compared to SPSS, it is not necessary to click at single objects in order to change its properties which could become inconvenient for complex figures or series of figures.

SPSS offers lots of different statistical tests regarding survival time analysis. In contrast, KMWin provides only the Logrank test, but allows adjustment of p-value accuracy. The Logrank test is most often used in survival analyses. KMWin can display number of cases and number of cases under risk at certain time points within the figure, which is not possible with SPSS but often required for publications.

Both programmes facilitate interim analyses and re-evaluation of updated data sets. In SPSS scripting is required to analyse data sets multiple times. Using KMWin, it is only needed to put saved plots into the File queue and choose the desired graphical output format. It is also straightforward to adapt single plots, i.e. open a saved plot, adapt it quickly and create a new figure.

SPSS and KMWin differ clearly regarding the interaction of programmer and user. SPSS provides lots of different statistical methods to many users. This makes it nearly impossible to fulfil specific requirements or to solve problems individually. KMWin is not as complex as SPSS and therefore the development is more straightforward allowing inclusion of desired features and requirements quickly.

Although the functional range of SPSS is larger in general, the functionality of KMWin is more appropriate for the requirements of our study-groups. KMWin can be used alone or in addition to SPSS.

Beside the commercial software packages, other free software is available which facilitate Kaplan-Meier survival time analysis, e.g. Gnumeric [8]. However, KMWin was designed in close cooperation with our biometricians, trials assistants and data managers, optimizing the software with respect to their needs and preferences. KMWin is currently used as a standard-tool for the analysis of a large number of multi-centre trials supported by our institute and the Clinical Trial Centre Leipzig. In our practice, KMWin was applied to generate about 170 figures provided for more than 20 PubMed listed publications. For examples, see [9]–[11]. Even more figures were prepared for numerous presentations at congresses or meetings of the study-groups.

In conclusion, our software tool does not provide the full functionality of commercial software packages but features a number of useful options for Kaplan-Meier survival analyses not available by other packages. These options comprise for example a queue system for repetitive analyses of interim data and statistics about number of cases under risk within the figure. It can be handled easily also by non-statisticians. The software is routinely applied in the Clinical Trial Centre Leipzig, in the German High-Grade Non-Hodgkin’s Lymphoma study group (DSHNHL) and our institute (IMISE). Therefore, we believe that it could be useful for other clinical trials centres as well.

## Methods

### Basic Concepts

KMWin makes use of two concepts, it uses functions of R exploiting the broad functionality of this software environment and it provides interface elements of Microsoft Windows [12].

When an appropriate data source with survival time data is given, KMWin uses R functions to conduct the analysis. Currently, KMWin supports SPSS files (sav), SAS xport files (xpt) and text files (dat) with white space (one or more spaces or tabs) separated columns. If survival time data are managed in other applications such as Excel, it can be exported as tab delimited text file.

Plots are generated in R and can be directly exported in any graphical output format supported by R which are Windows meta file (emf), Encapsulated postscript (eps), Portable document format (pdf), Portable network graphics (png), Bitmap (bmp), Tagged image file format (tif) and Joint photographic experts group file (jpg). All modifications regarding appearance, strata, filters and so on can be saved for future use. Meta data such as graphical settings, filter and location of the data source are stored in a (small binary) file and not the survival data itself.

KMWin internally manages fragments of R instructions, controlling establishment of the connection to R, the assignment of R variables and the call of R functions. These fragments are assembled according to the user input and sent to R. R variables are used for keeping the content of the data source and interim results. R functions are either defined by KMWin and applied for drawing figure elements (legend, p-value table, table of cases under risk, axes) or invoked to load required libraries, load the survival time data into R, do the survival time analysis or draw the results with user’s style preferences. Interface elements are managed by Windows API (Application Programming Interface) calls from the programme. The latest version 1.51 of KMWin was compiled with lcc-win32 C development environment version 3.8 [13]. R [3], [4] version 2.14.0 was used for analysis of the example data provided.

### Limitations

Many different libraries are available for R. This makes R a powerful and versatile software environment and well suited for statistical analysis. However, using R as basis for KMWin has some drawbacks. Interaction with the KMWin interface results in assembling appropriate fragments of R code, which control the survival time analysis. The user has not to deal with R script commands, but the static implementation of R instructions into KMWin makes it difficult to adapt the communication between both programmes in case of changed syntax of R functions. Instead, it would be possible to implement editable templates.

Furthermore, KMWin supports survival time data stored in SPSS and SAS files. Loading such files into R may cause problems too, particularly with respect to the way how variable names of the data source are handled (e.g. uppercase or not) by R libraries. This depends on the version of the library. Since KMWin requires exact matches for variable names in saved plots, only the same version of R should be used for re-processing plots.

Although KMWin was developed for Microsoft Windows, it can be run under Linux with Wine [14]. Since file paths differ between Linux and Windows, R for Windows must be run with Wine too.

### Data Simulation

We provide sample data to briefly demonstrate the creation of Kaplan-Meier plots and the features of the programme. For this purpose, we simulated time to event data of four entities with N = 110, N = 120, N = 130 and N = 70 individuals, respectively. The event times are sampled from an exponential distribution (i.e. constant hazard) with rates 0.02, 0.02, 0.02 and 0.04. Censoring is simulated by sampling the time of a second event for each individual from an exponential distribution with rate 0.04. An observation is censored, if the second event occurs prior to the first event. We assigned group labels 1,2,3,3 to the individuals of our groups, i.e. we do in fact consider three different treatment groups. We also introduced labels 1,1,1,2 for filtering purposes. When analysing unfiltered data, the survival curves of group 1 and 2 are the same. However, the survival curve of group 3 declines faster due to the subset of N = 70 individuals with higher event rate. When we remove the latter by filtering, we obtain three similar survival curves arising from identically distributed event times.

### Availability and Future Directions

The KMWin Package S1 consists of the binary, an HTML help file and sample data as described in “Data simulation”. KMWin is written in C. The source code of the most recent version can be found in Package S2. The project homepage of KMWin is located at Sourceforge [15]. KMWin is developed for Microsoft Windows. The R software environment is required to successfully run KMWin. The KMWin package is distributed under the GNU General Public License version 3. For details, see [16].

The software is maintained and improved in cooperation with biometricians and trial assistants of our study groups.

## Supporting Information

Package S1 **KMWin package.** The rar archive contains the binary, HTML help file and sample data.
(RAR)

Package S2 **Source code.** The rar archive contains all sources to build KMWin.
(RAR)
